# Effect of mitochondrially targeted carboxy proxyl nitroxide on Akt-mediated survival in Daudi cells: Significance of a dual mode of action

**DOI:** 10.1371/journal.pone.0174546

**Published:** 2017-04-20

**Authors:** Gokul Variar, Tarun Pant, Apoorva Singh, Abinaya Ravichandran, Sushant Swami, Balaraman Kalyanaraman, Anuradha Dhanasekaran

**Affiliations:** 1 Centre for Biotechnology, Anna University, Chennai, Tamilnadu, India; 2 Department of Biophysics and Free Radical Research Centre, Medical College of Wisconsin, Milwaukee, Wisconsin; University of PECS Medical School, HUNGARY

## Abstract

Vicious cycles of mutations and reactive oxygen species (ROS) generation contribute to cancer progression. The use of antioxidants to inhibit ROS generation promotes cytostasis by affecting the mutation cycle and ROS-dependent survival signaling. However, cancer cells select mutations to elevate ROS albeit maintaining mitochondrial hyperpolarization (Δψm), even under hypoxia. From this perspective, the use of drugs that disrupt both ROS generation and Δψm is a viable anticancer strategy. Hence, we studied the effects of mitochondrially targeted carboxy proxyl nitroxide (Mito-CP) and a control ten carbon TPP moiety (Dec-TPP^+^) in the human Burkitt lymphoma cell line (Daudi) and normal peripheral blood mononuclear cells under hypoxia and normoxia. We found preferential localization, Δψm and adenosine triphosphate loss, and significant cytotoxicity by Mito-CP in Daudi cells alone. Interestingly, ROS levels were decreased and maintained in hypoxic and normoxic cancer cells, respectively, by Mito-CP but not Dec-TPP^+^, therefore preventing any adaptive signaling. Moreover, dual effects on mitochondrial bioenergetics and ROS by Mito-CP curtailed the cancer survival via Akt inhibition, AMPK-HIF-1α activation and promoted apoptosis via increased BCL2-associated X protein and poly (ADP-ribose) polymerase expression. This dual mode of action by Mito-CP provides a better explanation of the application of antioxidants with specific relevance to cancerous transformation and adaptations in the Daudi cell line.

## Introduction

Cancer is a metabolic disease, the metabolic alterations and proliferation of which are caused by oncogenic mutations and/or oncogenic viruses. Alterations within the cancer niche are not coordinated with the surrounding normal cells; this affects their homeostasis [***1***]. Daudi, a model cell line for endemic Burkitt’s lymphoma, is strongly associated with the Epstein Barr virus (EBV), an oncogenic virus. Each Daudi cell contains about 80 copies of EBV. The immortalization of normal B-cells into the Daudi cell line involves the cellular transformation termed Latency III [***2,3***]. This is mediated by EBV proteins, including five nuclear proteins (EBNA-1, -2, -3, -5, and -6) and one latent membrane protein (LMP-1) [***4,5***]. For example, Epstein-Barr nuclear antigen 1 (EBNA 1), a regularly detected protein of all EBV-associated malignancies, transcriptionally activates the expression of nicotinamide adenine dinucleotide phosphate-oxidase (NADPH) oxidase 2 (NOX2), a key generator of reactive oxygen species (ROS) that is elevated under Latency III [***6,7***]. This elevated ROS response mediated by EBV promotes activation of Akt, by oxidation-reduction (redox) inhibition of its phosphatase (PP2A), and helps to counteract apoptosis and favor cell survival. This is highlighted by the fact that ROS scavengers (such as N-acetyl-L-cysteine [NAC], catalase, and reduced glutathione) have been shown to have positive effects in inhibiting EBV reactivation [***8***]. Moreover, EBNA-5 and -3 have been shown to bind and inhibit prolyl hydroxylases 1 and 2, respectively. This leads to activation of hypoxia-inducible factor 1-alpha (HIF1-α) and results in a metabolic reprogramming associated with HIF1-α responsive genes (such as Glucose transporter 1 [GLUT1], phosphofructokinase [PFK], lactate dehydrogenase enzyme 1 [LDHA1], monocarboxylate transporter 4 [MCT4] [solute carrier family 16 member, 3SLC16A3], and pyruvate dehydrogenase lipoamide kinase isozyme 1 [PDK1]), which eventually favor aerobic glycolysis even under normoxic conditions [***9***]. Thus, the net adaptive strategies of EBV-transformed Daudi cells involve key cellular mediators Akt and HIF1-α, of which the former lies in the upstream regulation of the later. A promising strategy to divert EBV cellular immortalization in Daudi cells towards apoptosis is to target Akt activation through the cell-specific delivery of antioxidants.

In the context of specificity, the endogenous mode of specific antiviral response via type 1 interferon has been shown to decrease the activities of cytochrome c oxidase and NADH (nicotinamide adenine dinucleotide)-cytochrome c reductase, leading to the inhibition of mitochondrial electron transport and, hence, decreasing cellular adenosine triphosphate (ATP) levels in the Daudi cells hindering its proliferation. This highlights the importance of mitochondrial bioenergetic’s influence on the proliferation of Daudi cells, as well as cancers derived from oncogenic mutations [***10***]. Interestingly, cancer cells maintain optimal mitochondrial electron transport chain (ETC) function, even under oxygen level conditions as low as 0.5%. Maintenance of a distinct hyperpolarized state of mitochondria under both normoxic and hypoxic conditions offers an attractive strategy to selectively delivering antioxidant moieties into cancer cells [***11,12***]. As such, we investigated the mode of action of a mitochondrial membrane potential dependent antioxidant (Mito-CP) on cancerous Daudi cells and normal peripheral blood mononuclear cells (PBMCs). Moreover, since localized hypoxia in various tissues (such as lymph node, colon, and liver) promotes organ-specific homing and tumor spreading [***13***], we were also keen to understand the effect of Mito-CP under hypoxic conditions.

In the present study, we found that Mito-CP treatment under hypoxia and normoxia induces significant high toxicity and apoptosis in Daudi cells than in PBMCs. Further, we found the increase in apoptosis with Mito-CP treatment under hypoxia and normoxia was associated with increased mitochondrial localization, and decreased mitochondrial membrane potential and ATP production in Daudi cells than in PBMCs. Interestingly, ROS levels decreased and were maintained in hypoxic and normoxic Daudi cells, respectively, therefore preventing any adaptive signaling via Akt. Regulation of ROS, ATP, Akt and AMPK by Mito-CP was accompanied by a decrease in hypoxia-inducible factor 1-alpha (HIF-1α) and an increase in apoptotic markers such as poly(ADP-ribose) polymerases (PARP) and cytochrome C (cyt c) in Daudi cells than in PBMCs. Hence, this study elucidates that Mito-CP is an effective chemotherapeutic agent whose selective localization induces apoptosis in Daudi cells, than in PBMCs, by a dual-mode mechanism involving its effects on mitochondrial bioenergetics and antioxidant effects that cumulatively prevent survival through Akt signaling. To validate Mito-CP’s use for anticancer therapy, its dual anticancer activity mechanisms can be further evaluated in vivo and in cancers with similar survival strategies.

## Materials and methods

### Drug and cell culture

Mito-CP was obtained from Dr. Balaraman Kalyanaraman’s lab (Department of Biophysics and Free Radical Research Center, Medical College of Wisconsin, Milwaukee, USA), with whom we are collaborating on the present work. The hypoxia chamber was purchased from Eppendorf India (Chennai, India). Daudi cells (human B lymphoblast cells) were purchased from the National Centre for Cell Science (Maharashtra, India) and maintained in Gibco RPMI-1640 (Life Technologies, Grand Island, New York, USA) medium with 100 U penicillin/ml (HiMedia Laboratories, LBS Marg, Mumbai, India), 100 μg streptomycin/ml (HiMedia Laboratories, LBS Marg, Mumbai, India) and 10% fetal bovine serum (FBS) (Life Technologies, Grand Island, New York, USA), Dec-TPP^+^ (Sigma Aldrich, St.louis, Missouri.)

The study has been approved by the Anna University Institutional Ethical Review Board (AU- IERB) and Anna University Institutional Biosafety Committee (AU-IBSC) Centre for Biotechnology, Anna University, Chennai, Tamilnadu, India and all the subjects gave written informed consent. Blood used to isolate PBMC was collected from healthy human volunteers using a heparinized syringe. PBMC was isolated from 20 mL of normal human blood by HiSep LSM 1077 Ficoll solution (HiMedia Laboratories, LBS Marg, Mumbai, India) taken in a sterile Falcon tube. Blood was slowly added to the Ficoll solution tube, in a slanted position, in a 2:1 ratio. Cells were spun at 448 g for 20 min. at 4°C. Buffy coat was removed carefully and collected in a fresh Falcon tube. Again, the cells were spun at 448 g for 15 min. at 4°C. The supernatant was discarded, and the pellet was washed in 5 mL of incomplete RPMI medium (without FBS). Cells were spun at 252 g for 10 min, and the supernatant was discarded and resuspended in 1 mL of completed RPMI medium with 10% FBS. Cells were counted by taking 10 μL of cells and 10 μL of Gibco trypan blue stain (Life Technologies, Grand Island, New York, USA) in a haemocytometer [***14***].

### Cell treatment

On the day of treatment, Daudi cells and PBMCs were grown to 1x10^6^ cells/ml in a T-25 flask, centrifuged and resuspended in RPMI medium with and without 1 μM concentration Mito-CP under normoxia (21% oxygen [O_2_]) in an incubator with 5% carbon dioxide (CO_2_) at 37°C for intervals of 6 h and 24 h respectively. Cells were analyzed before and after Mito-CP treatment. For hypoxia, Daudi cells and PBMCs were incubated in the hypoxia chamber with 5% O_2_, 5% CO_2_ at 37°C with and without 1 μM Mito-CP for time intervals of 6 h and 24 h respectively. Daudi cells and PBMCs were also subjected to treatment with Dec-TPP^+^ 1μM concentration under normoxia and hypoxia.

### Cell viability

Cell viability was determined by using the alamarBlue reagent (Invitrogen, Frederick, MD, USA) to detect the metabolic activity of the cells. AlamarBlue incorporates a redox indicator phenothiazine that accelerates the reduction process of resazurin to resorufin which fluoresces and changes color in presence of intracellular reductants such as (NADH, FADH, FMNH). A negative control was taken in triplicate in a flat-bottom 96-well plate containing medium only and no cells. Daudi cells and PBMCs (approximately 3,000 cells per well) were taken in a 96-well plate. Similarly, cells treated with and without Mito-CP (1 μM) and subjected to hypoxia were also analyzed for viability by adding 10 μL of the alamarBlue reagent to all the wells and incubating them for 8 h. Absorbance was read at 570 nm in an enzyme-linked immunosorbent assay (ELISA) plate reader (BioTek Instruments, Winooski, VT, USA).

### Cell proliferation

The cells’ proliferation rate was analyzed with the alamarBlue reagent (Invitrogen, Frederick, MD, USA). Each well of 96-well plate was seeded with 3,000 cells, and all were subjected to hypoxia with and without Mito-CP treatment. Cells after treatment were incubated for 24 h. Then, 10 μL of the alamarBlue reagent was added and the cells were incubated for an additional 8 h. Absorbance was read at 570 nm in an ELISA plate reader.

### Apoptosis assay using Annexin V-FITC and propidium iodide

Daudi cells and PBMC’s were grown to and subjected to treatment under hypoxia and normoxia with and without Mito-CP (1 μM), as described previously. Suspension cells were centrifuged, and the pellet was washed twice with PBS (phosphate-buffered saline). Cell density was determined and diluted to approximately 4x 10^5^ cells/ml and stained with a combination of 10 μL Annexin V-FITC conjugate and 1 μL of propidium iodide according to the kit protocol (Molecular Probes, Eugene, OR, USA), and the fluorescent images were scanned using confocal laser scanning microscopy. The co-localization fluorescence intensity of both annexin V-FITC and propidium iodide were quantified using image J software.

### ATP production assay

Cellular ATP content was analyzed to determine the bioenergetics of the Daudi and normal PBMC’s. Cells subjected to treatment of hypoxia, with and without treatment of Mito-CP, were seeded, and intracellular ATP levels were determined using a bioluminescence-based assay as per the manufacturer’s protocol (Molecular Probes, Eugene, OR, USA). Luminescence were measured by luminescence plate reader (BioTek Instruments, Winooski, VT, USA).

### Detection of reactive oxygen species

ROS (hydrogen peroxide) release was measured with an Amplex Red reagent (Molecular Probes, Eugene, OR, USA) according to the manufacturer’s protocol. Cells subjected to hypoxia, with and without treatment of Mito-CP, and 20 μl of approximately 2x 10^4^ Daudi and PBMC’s suspended in Krebs ringer phosphate buffer were added to a 96-well plate along with 100 μL of reaction mixture containing 50 μM Amplex Red reagent and 0.01 uHRP/mL in a Krebs ringer phosphate buffer and incubated at 37°C for 10 min. Readings were taken in an ELISA plate reader at 560 nm.

### Mitochondrial membrane potential

Daudi cells and PBMCs were seeded in a 96-well plate at a density of 6×10^4^ cells per well in 100 μL medium in triplicate. Cells were treated under hypoxia and normoxia without and without Mito-CP. After treatment, 10 μL JC-1 dye solution (Cayman Chemicals, USA, catalogue no. 10009908) was added to each well and cells were incubated for 30 min. Next, cells were washed twice with an assay buffer by centrifuging at 400g for 5 min. and resuspended in a 200 μL assay buffer according to the manufacturer’s protocol. Then, the plate was read for fluorescence intensity using a fluorescent plate reader with excitation and emission, respectively, at 535 nm and 595 nm for J aggregates and 485 nm and 535 nm for J monomers.

### EPR of mitochondrial localization of Mito-CP

Mitochondria were isolated from Daudi cells and PBMCs with Mito-CP (1 μM) treatment under hypoxia and normoxia. After treatment, cells were washed in 1×TBS and centrifuged at 600 g for 10 min at 4°C. The pellet was suspended in mitochondrial isolation buffer (I_B_) (10 ml of 0.1 Tris-MOPS [3-(N-morpholino)propanesulfonic acid], 1 ml of EGTA [ethylene glycol-bis(β-aminoethyl ether)-N,N,N',N'-tetraacetic acid]/Tris, and 20 ml of 1M sucrose) and disrupted in a sonicator (20 hz frequency in 10 30-sec. cycles). The homogenate was centrifuged at 600 g for 10 min. at 4°C, and the mitochondria were isolated according to Ref. [***15***]. Finally, the pellet containing mitochondria was suspended in a mitochondrial isolation buffer. Electron Paramagnetic Resonance/Electron Spin Resonance (EPR/ESR) data were acquired with a JEOL JES-FA200 electron spin resonance spectrometer at the Indian Institute of Technology facility in Madras, Chennai. The parameter and EPR settings used in EPR spectra are as follows: G_xx_ = 2.0089, G_yy_ = 2.0058, G_zz_ = 2.0021, A_xx_ = 5.6, A_yy_ = 5.3, A_zz_ = 34 G, β = 60°, R_xx_ = 8.9×10^7^, r_yy_ = 8.9x10^7^, r_zz_ = 1.0x10^7^s^-i^, Ψ = 60°, C_20_ = 2.00 [***16***].

### Detection of protein by western blotting

Daudi cells and PBMCs were treated under hypoxia and normoxia with and without Mito-CP. Cells were lysed using 1×RIPA buffer, and 1 mm PMSF (phenylmethylsulphonylfluoride) was added; then, the solution was kept on ice for 5 min. The cell lysates were centrifuged at 14,000 g for 10 min. in a cold centrifuge, and supernatant was stored at -20°C after aliquoting as a small working-stock solution. Protein concentration was measured by the Bradford method. Aliquots of the lysate (40 μg of protein) were separated on 10% w/v sodium dodecyl sulfate-polyacrylamide gel and transferred onto a nitrocellulose membrane with a glycine transfer buffer (192 mM Glycine/25 mM Tris–hydrochloride [ph = 8.8]/20% methanol [v/v]). The membrane was transferred to 5% bovine serum albumin (BSA) in 1×TBS, to block the nonspecific site, and incubated overnight in a rocker at 4°C. The various primaries used include rabbit monoclonal antibody Akt, p-Akt, AMPK, p-AMPK, the x-linked inhibitor of apoptosis protein (XIAP), Cytochrome c, cleaved PARP, and β-actin (Cell Signaling Technologies, Danvers, MA, USA), HIF-1α (Santa Cruz Biotechnology, Dallas, TX, USA). The membrane was incubated in a primary antibody solution (1:1000 dilution in a primary dilution buffer) overnight. Then, the membrane was washed thoroughly in 1×TBST and TBS for 30 min to remove any nonspecific bound antibodies. The membrane was incubated in a secondary antibody goat anti-rabbit IgG alkaline phosphatase (for HIF-1α) and goat anti-rabbit IgG horse radish peroxidase (for the other proteins) (Santa Cruz Biotechnology, Dallas, TX, USA) for 45 min. For developing, chromogenic NBT/BCIP (nitro blue tetrazolium/5-bromo-4-chloro-3-indolyl-phosphate) (Thermo Fisher Scientific, Rockford, IL, USA) for alkaline phosphatase secondary antibody and ECL (enhanced chemiluminescence) (GE Healthcare, Amersham, UK) for HRP-conjugated secondary antibody were used. The band intensities of individual proteins were quantified and normalized with corresponding β-Actin band intensity using Image lab and Image studio lite ver. 5.2 software and arbitrary units with respect to control groups are represented as bar graphs.

### Quantitative real-time polymerase chain reaction

Pro-apoptotic gene BCL2-associated X protein (BAX) was determined by RT-PCR (reverse transcription polymerase chain reaction). Total RNA (ribonucleic acid) was isolated from the Daudi cells and PBMCs under hypoxia and normoxia with and without Mito-CP using a Trizol reagent (Bangalore GeNei by Merck, Darmstadt, Germany). Primer-BLAST software was used to design the primers glyceraldehyde 3-phosphate dehydrogenase (GAPDH) (Sigma-Aldrich, St. Louis, MO, USA) (sense 5^’^-3^’^ CCACCCATGGCAAATTCCATGGCA and antisense 5^’^-3^’^ TCTAGACGGCAGGTCAGGTCAACC) and BAX (Sigma-Aldrich, St. Louis, MO, USA) (sense 5^’^-3^’^ TTCATCCAGGATCGAGCAGG and anti-sense 5^’^-3^’^ GGAAAAAGACCTCTCGGGGG). GAPDH was taken as an internal control. cDNA was mixed with SYBR Green Master (Roche Diagnostics, Indianapolis, IN, USA), and the reaction volume was brought up to 10 μL with PCR-Grade water (Sigma-Aldrich, St. Louis, MO, USA) and analyzed using the Applied Biosystems StepOne Real-Time PCR instrument. Amplified products were analyzed using a melting curve analysis for each primer pair, and comparative threshold cycle data values were noted. Data were then analyzed for a fold change in expression using the formula 2^-ΔΔCT^.

### Statistical analysis

All data were presented as mean ± standard error (SEM) and repeated three to five times in each experiment independently. The statistical differences between groups were analyzed by two way ANOVA (analysis of variance) with Bonferroni Post-test in graph pad prism 5 software. P < 0.05 and P < 0.01 were considered statistically significant.

## Results

### Cytotoxic, antiproliferative and apopototic effects of Mito-CP in Daudi Cells and PBMCs

**Fig 1:**
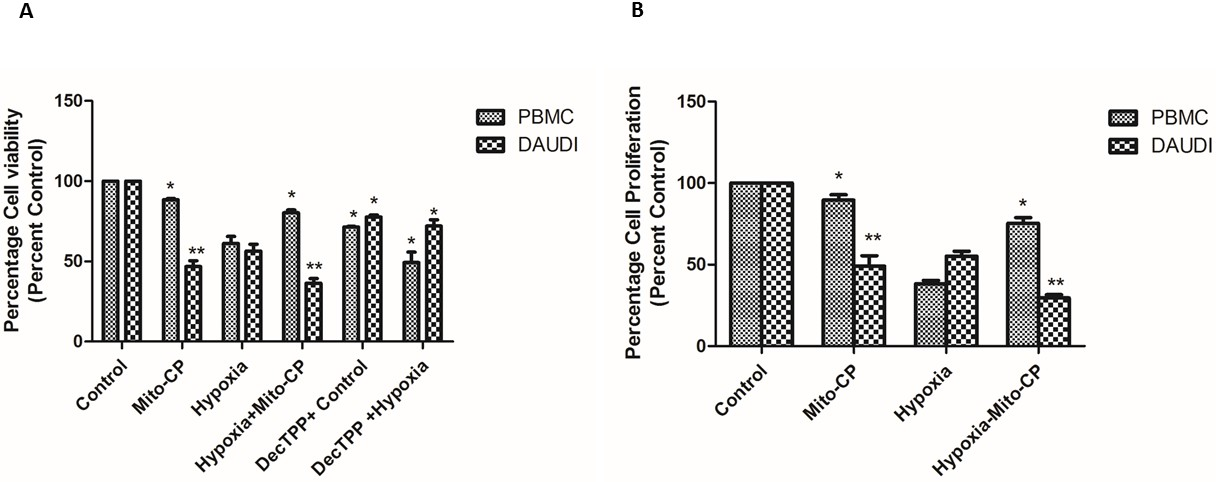
Effect of *Mito-CP on cell viability and cell proliferation in Daudi cells and PBMCs by alamarBlue assay*. Daudi cells and PBMC were treated with and without Mito-CP under hypoxia (5% O_2_) and normoxia. **(*A*)** Shows percentage of cell viability after 6 h in Daudi and PBMC treated with and without Mito-CP and Dec-TPP^+^ under hypoxia and normoxia. Bar graph plotted represents percentage of viable cells normalised value to percent control. Data were obtained from three separate experiments and were expressed as by mean ± SEM. * and ** denotes significantly different compared to control p<0.05 and p<0.01 respectively. **(*B*)** Shows percentage of cell proliferation after 24 h in Daudi and PBMC treated with and without Mito-CP under hypoxia and normoxia. Each bar graph represented was normalised to percent control. Data were obtained from three separate experiments and are expressed as mean ± SEM. * and **, significantly different compared to control p<0.05 and p<0.01 respectively.

**Fig 2:**
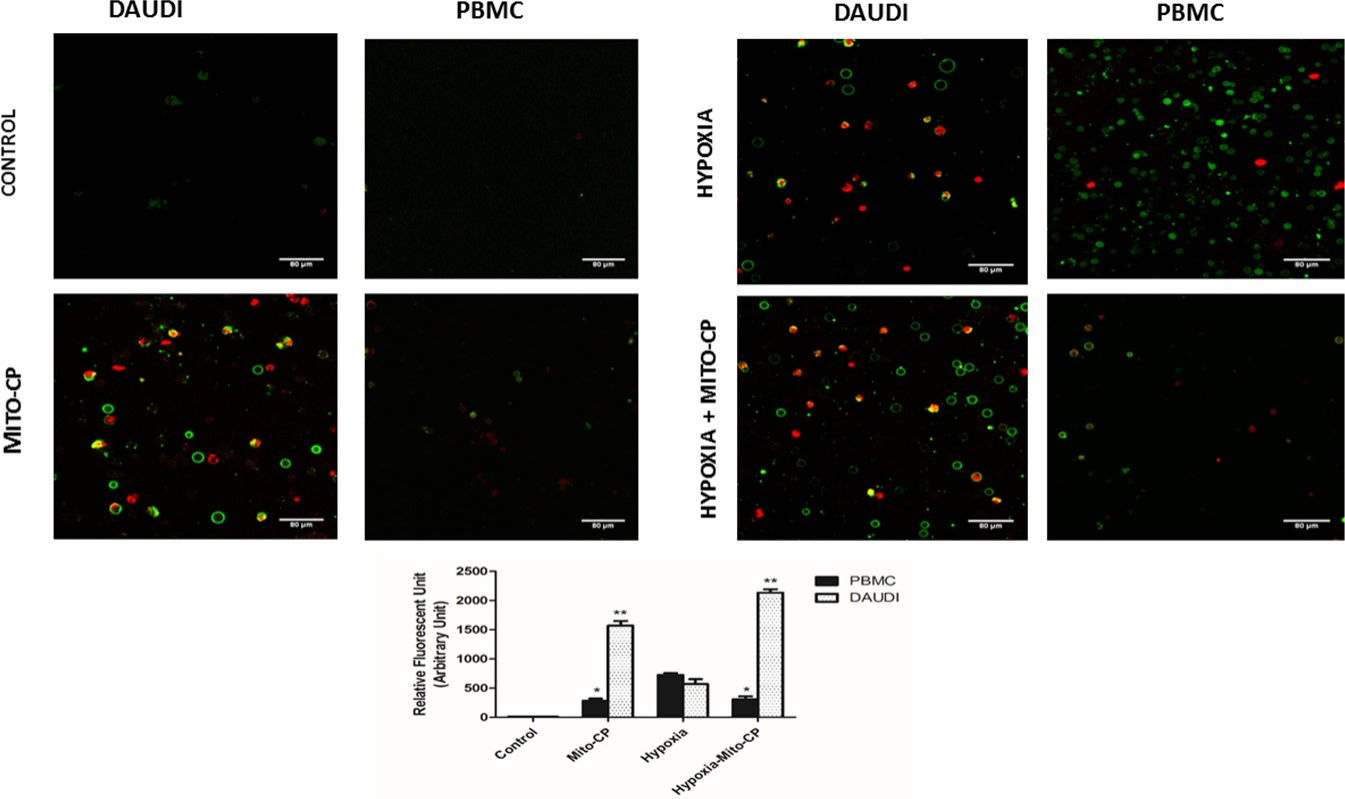
Effect of Mito-CP induced apoptosis by Annexin V-FITC staining. Characteristic phenomenon of Phosphotidyl serine externalisation and disruption in cell membrane in apoptotic cells were observed by staining cells with Annexin V FITC and Propidium Iodide. Daudi cells and PBMCs were treated with and without Mito-CP (1μM) under hypoxia (5% O_2_) and normoxia for a period of 6 h. Cells were visualised under confocal laser scanning microscope and photographed. Images obtained were analysed by Image J software and the data is expressed as bar graph. Data were obtained from five different experiments and are expressed as mean ±SEM. * and **, significantly different compared to control p<0.05 and p<0.01 respectively.

AlamarBlue dye was used to analyze cell viability and proliferation in Daudi cells and PBMCs. Daudi cells treated with Mito-CP showed a significant decrease 54% and 64% (P < 0.01) in cell viability under normoxia and hypoxia respectively “***Fig 1A***”. PBMCs treated with Mito-CP also showed a significant but less loss (12%—P<0.05) in cell viability under normoxia. Moreover, Mito-CP showed a significant protection against hypoxia induced decrease in cell viability in PBMC. In comparison to Mito-CP, Dec-TPP^+^ alone treatment under hypoxia and normoxia in Daudi showed a weaker decrease in cell viability (28% and 23% respectively–P<0.05) in Daudi cells “***Fig 1A***”. Also Dec-TPP^+^ treatment alone in PBMC’s under hypoxia and normoxia showed a significant (P<0.05) cytotoxicity. A similar effect (P<0.01) with regard to anti-proliferative effects in Daudi cells with Mito-CP treatment under hypoxia and normoxia was observed “***Fig 1B***”. On the other hand, PBMCs treated with Mito-CP showed less significant decrease (P<0.05) in cell proliferation under normoxia than Daudi cells and significant (P<0.05) protection of cell proliferation under hypoxia “***Fig 1B***”. Mito-CP induced apoptosis was analyzed with Annexin V-FITC and Propidium iodide staining. Daudi cells treated with Mito-CP showed significantly increased (P<0.01) Annexin V and positive cells under normoxia and hypoxia. Annexin V positive cells were stained with Propidium iodide which confirms the presence of dead or late apoptotic cells. PBMCs treated with Mito-CP showed a less significant increase (P<0.05) in Annexin V positive cells under hypoxia and normoxia “***Fig 2***”.

### Mitochondrial membrane potential, ATP, ROS and localization effects of Mito-CP

**Fig 3:**
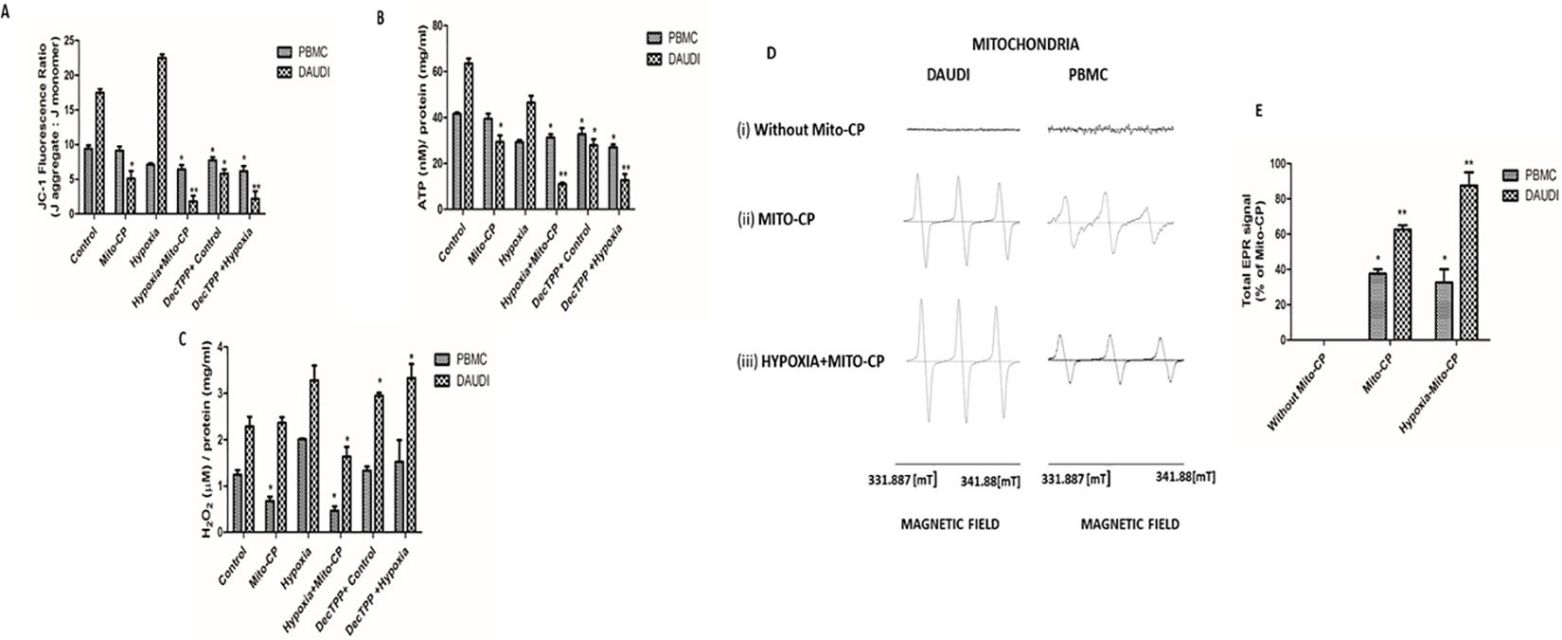
Effect of Mito-CP on mitochondrial membrane potential, ATP levels, Hydrogen peroxide levels and subcellular mitochondrial localisation. ***(A)*** Mitochondrial membrane potential in Daudi cells and PBMCs with and without Mito-CP (1μM) and Dec-TPP^+^ (1μM) under hypoxia (5%O_2_) and normoxia were measured using JC-1 dye. Data were obtained from three separate experiments and are expressed as mean ± SEM. * and **, significantly different when compared to control p<0.05 and p<0.01 respectively. (***B***) Cellular ATP levels in Daudi cells and PBMC were determined using a bioluminescence based assay kit with or without Mito-CP (1μM) and Dec-TPP^+^ (1μM) treatment under hypoxia (5%O_2_) and normoxia. ATP concentration were determined using the standard ATP provided with the manufacturer’s kit (Molecular Probes, Eugene, OR, USA). Bar graph represented was normalised to protein concentration. Data were obtained from three separate experiments and are expressed as mean ± SEM. * and **, significantly different when compared to control p<0.05 and p<0.01 respectively. (***C***) Hydrogen peroxide levels in Daudi cells and PBMCs with and without Mito-CP (1μM) and Dec-TPP^+^ (1μM) under hypoxia (5%O_2_) and normoxia were measured using Amplex red assay. Concentration of hydrogen peroxide were obtained using the standard hydrogen peroxide provided with the amplex red reagent manufacturer’s kit (Molecular Probes, Eugene, OR, USA). Bar graph represented was normalised to protein concentration. Data were obtained from three separate experiments and are expressed as mean ± SEM. *, significantly different when compared to control p<0.05. (***D***) Shows EPR monitoring of mitochondrial localization of Mito-CP in Daudi cells and PBMC under hypoxia (5%O_2_) and normoxia. ***(i)*** EPR spectra were obtained from mitochondrial fraction of Daudi cells and PBMCs treated with and without Mito-CP. ***(ii)*** As was done for (i), Daudi cells and PBMCs were treated with Mito-CP (1μm). ***(iii)*** As was done for (i), Daudi cells and PBMCs were treated with Mito-CP under hypoxia. The parameters used in EPR spectra follow: G_xx_ = 2.0089, G_yy_ = 2.0058, G_zz_ = 2.0021, A_xx_ = 5.6, A_yy_ = 5.3, A_zz_ = 34 G, β = 60°, R_xx_ = 8.9×10^7^, r_yy_ = 8.9x10^7^, r_zz_ = 1.0x10^7^s^-i^, Ψ = 60°, C_20_ = 2.00. (***E***) Shows quantified data of total EPR signal intensity of Mito-CP in Daudi cells and PBMC under normoxia and hypoxia. Concentration of cell lysate used for analysis was determined by Bradford method and equal amount of concentration in each sample was analysed in EPR spectrometer. EPR signal intensity was normalized to the peak intensity obtained by Mito-CP alone before treatment. Data were obtained from three separate experiments and are expressed as mean ± SEM. * and ** denotes significantly different when compared to control p<0.05 and p<0.01 respectively.

Analysis of mitochondrial membrane potential using JC-1 dye showed an elevated mitochondrial membrane potential in Daudi cells under normoxia and hypoxia. Mito-CP treatment caused a significant decrease (P<0.01 and P<0.05) in mitochondrial membrane potential in Daudi cells under hypoxia and normoxia. In PBMCs Mito-CP treatment did not cause any significant decrease in membrane potential under normoxia but caused a less significant decrease (P<0.05) under hypoxia. Dec-TPP^+^ treatment caused a significant decrease (P<0.05 and P<0.01) in membrane potential in Daudi cells under normoxia and hypoxia and a less significant (P<0.05) membrane potential decrease in PBMC’s “***Fig 3A***”. Daudi cells treated with Mito-CP showed a significant decrease (P<0.01 and P<0.05) in ATP levels under hypoxia and normoxia than PBMC’s under normoxia. Dec-TPP^+^ treatment in Daudi cells also showed a significant decrease P<0.05 and P<0.01 in ATP levels under normoxia and hypoxia. “***Fig 3B***”. PBMCs treated with Mito-CP did not show a significant decrease in cellular ATP levels under normoxia and a significant (P<0.05) increase in ATP levels under hypoxia. PBMCs treated with Dec-TPP^+^ showed a significant ATP level decrease (P<0.05) under normoxia and hypoxia “***Fig 3B*”.** Study on cellular ROS (H_2_O_2_) levels showed that Daudi cells have elevated H_2_O_2_ levels under hypoxia compared to normoxia. Moreover, Daudi cells treated with Mito-CP showed a significant (P< 0.05) decrease in H_2_O_2_ levels under hypoxia and a fairly insignificant decrease under normoxia. PBMCs under hypoxia showed a significant increase in H_2_O_2_ levels compared to normoxia but when treated with Mito-CP PBMCs showed a significant decrease (P<0.05) in H_2_O_2_ levels, both under hypoxia and normoxia. H_2_O_2_ levels in Daudi cells under Dec-TPP^+^ treatment were rather found to be significantly increased (P<0.05) under normoxia and hypoxia. PBMCs on treatment with Dec-TPP^+^ showed no significant changes in H_2_O_2_ levels between normoxia and hypoxia treatments. “***Fig 3C***”. An EPR spectra analysis was performed to determine the localization of Mito-CP in isolated mitochondria of Daudi cells and PBMCs, as the accumulation of Mito-CP correlates with the EPR signal intensity [**16**]. The EPR spectra analysis showed that there was no intensity in untreated Daudi cells and PBMCs. Further, it showed that, after 6 h of treatment, the signal intensity of Mito-CP was significantly higher (P<0.01) in Daudi cells than in PBMCs. Similarly, under hypoxia, the signal intensity of Mito-CP observed in the mitochondria of Daudi cells was significantly higher (P<0.01) than in the mitochondria of PBMCs, which proves there was much less accumulation in the PBMCs “***Fig 3D and 3E***”.

### Effect of Mito-CP on pro-apoptotic BAX mRNA levels and HIF-1α protein levels

**Fig 4:**
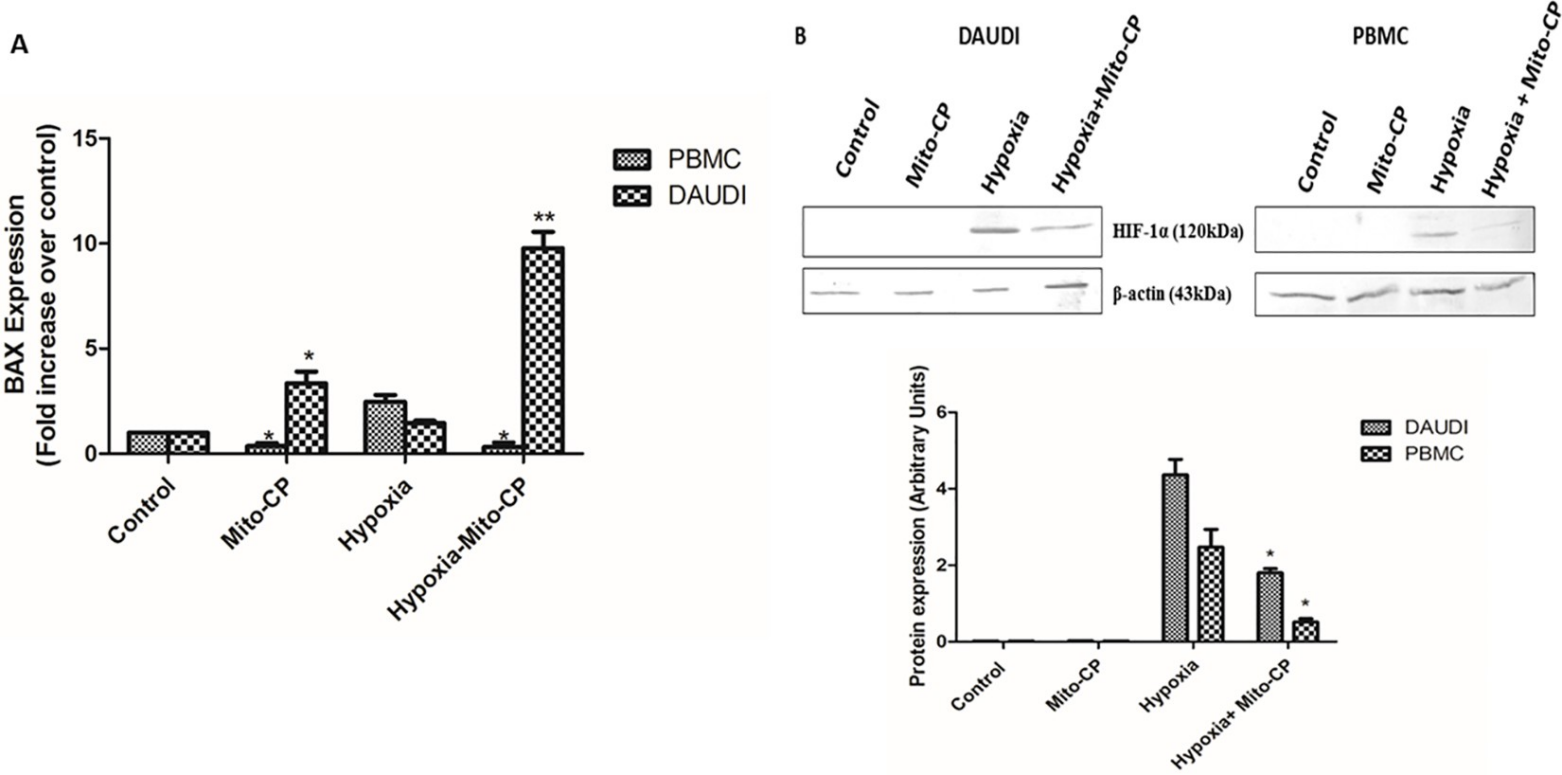
Effect of Mito-CP on BAX mRNA expression levels and HIF-1α protein expression levels. ***(A)*** Real time polymerase chain reaction were performed to quantify BAX mRNA levels in Daudi and PBMC with and without Mito-CP (1μM) treatment under hypoxia (5%O_2_) and normoxia. Amplified BAX mRNA was analysed by melting curve analysis and fold change in expression in each experimental group were calculated by 2^-ΔΔCT^. Data were obtained from three separate experiments and are expressed as mean ± SEM. * and ** denotes significantly different when compared to control p<0.05 and p<0.01 respectively. (***B***) Daudi cells and PBMCs were treated with and without Mito-CP (1μM) under hypoxia (5%O_2_) and normoxia. HIF-1α levels were measured by western blot and densitometric analysis are shown as bar graphs. Data were obtained from three separate experiments and were expressed as by mean ± SEM. * denotes significantly different when compared to control p<0.05.

The expression levels of pro-apoptotic BAX mRNA, in Daudi cells, increased tenfold under hypoxia and increased significantly (P<0.05) under normoxia after Mito-CP treatment. However, in PBMCs, the expression levels in BAX mRNA decreased significantly (P<0.05) after treatment with Mito-CP, under both hypoxia and normoxia “***Fig 4A***”. Protein expression of HIF-1α in Daudi cells was analyzed by western blot. Both in Daudi cells and PBMC, the HIF-1α protein was expressed only in hypoxia but not in normoxia and Mito-CP treatment under hypoxia caused a significant decrease (P<0.05) in HIF-1α in both the cells when normalized to cellular β-Actin content “***Fig 4B***”.

### Effects of Mito-CP on Akt mediated survival signaling and apoptotic markers

**Fig 5:**
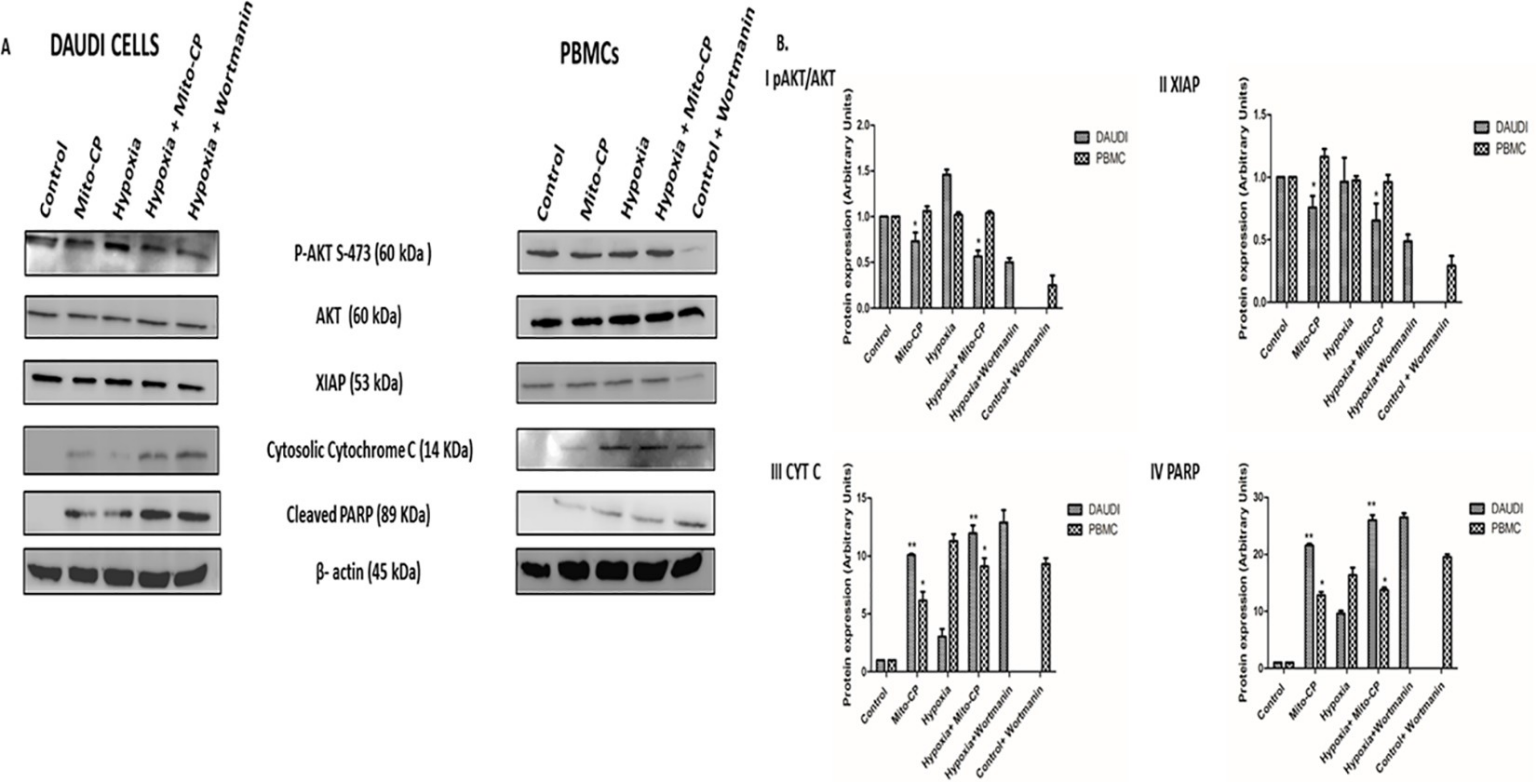
Effect of Mito-CP on AKT signaling pathway and apoptotic marker protein expression levels. ***(A)*** Daudi cells and PBMC were treated with and without Mito-CP (1μM) under hypoxia (5%O_2_) and normoxia. AKT inhibitor wortmanin (1μM) was also used to show inhibition of p-AKT. Protein lysate concentration was determined by Bradford method. P-AKT, XIAP, cytochrome c, cleaved PARP were measured by western blot. β-actin was used to normalise of protein expression. (***B***) Shows densitometry analysis of p-AKT, XIAP, cytochrome c, cleaved PARP. Data were obtained from three separate experiments and were expressed as by mean ± SEM.

**Fig 6:**
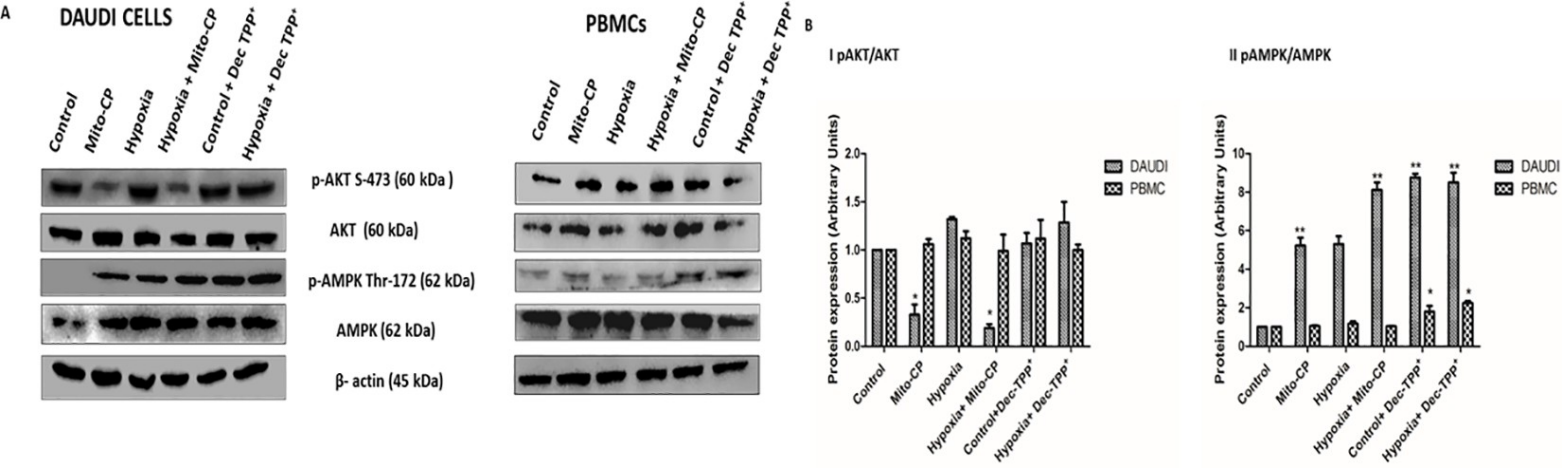
Comparative effect of Mito-CP and Dec-TPP^+^ on AKT and AMPK expression levels. (***A***) Daudi cells and PBMC were treated with and without Mito-CP (1μM) and Dec-TPP^+^ (1μM) under hypoxia (5%O_2_) and normoxia. P-AKT, AKT, P-AMPK, AMPK were measured by western blot. Β-actin was used to normalise of protein expression. (***B***) Densitometry analysis of P-AKT, AKT, P-AMPK, and AMPK were performed and the beta actin normalized P-AKT/Total-AKT and P-AMPK/Total-AMPK values were represented as bar graph. Data were obtained from three separate experiments and were expressed as by mean ± SEM. * and **, significantly different when compared to control p<0.05 and p<0.01 respectively.

Western blots were used to analyze p-Akt, Akt, XIAP, PARP, and cytochrome C protein expression in Daudi cells and PBMCs. In Daudi cells, p-Akt had high expression levels under hypoxia, was downregulated significantly (P<0.05) in Mito-CP treatment under hypoxia, and also was downregulated significantly (P<0.05) in Mito-CP treatment under normoxia. But, in PBMCs, p-Akt’s expression levels did not change under hypoxia or under normoxia in Mito-CP treatment, thus protecting PBMCs from apoptosis and enhancing survival. XIAP, the Akt pathway downstream protein, was regulated similarly to p-Akt in both Daudi cells and PBMCs. Apoptotic markers such as PARP and cytochrome C expression were significantly enhanced (P<0.01) with Mito-CP treatment in Daudi cells compared to PBMCs, under hypoxia and normoxia. The pathway expression was confirmed by blocking Akt signaling using the PI3K inhibitor wortmannin under hypoxia in Daudi cells and under normoxia in PBMCs “***Fig 5A and 5B***”. There was no effect on AKT phosphorylation levels by Dec-TPP^+^ treatment in Daudi under hypoxia and normoxia. In PBMCs Dec-TPP^+^ treatment did not show any significant decrease in AKT levels under normoxia and hypoxia. Dec-TPP^+^ treatment under normoxia and hypoxia showed a significant increase (P<0.01) in phospho-AMPK/total-AMPK expression levels in Daudi cells than in PBMC’s “***Fig 6A and 6B***”.

## Discussion

In this study, we have shown for the first time that the anticancer property of the mitochondrially targeted antioxidant Mito-CP in the Burkitt lymphoma Daudi cell line is mediated through its effects on mitochondrial bioenergetics and antioxidant properties. Mito-CP consists of an alkyl chain linking its antioxidant nitroxide moiety to the lipophilic and cationic triphenylphosphonium (TPP) moiety. The cationic TPP moiety is lipophilic and can freely pass through the phospholipid bilayer of the plasma membrane and other organelles, without the requirement for a specific uptake transporter protein. This property of Mito-CP can be exploited to overcome multidrug resistance evolved by cancer cells against various chemotherapeutic drugs by elevating p-glycoprotein pumping, and MDR and ABCB (ATP-linked drug transporter) protein expression [***17–22***]. The cells’ negative plasma membrane potential (−30 mV to −60 mV) drives the movement of TPP from the extracellular space into the cytoplasmic compartment up to tenfold. TPP can further accumulate within the mitochondria up to several hundredfold due to the higher mitochondrial membrane potential (−140 mV to −180 mV) [***23***]. It has been determined that the increase in TPP accumulation within the mitochondria is approximately tenfold for every 60-mV increase in mitochondrial membrane potential [***16***]. Compared with PBMCs, Daudi cells exhibit a higher membrane potential under normoxic and hypoxic states, with peak values under hypoxia; as such, a selective localization of Mito-CP in Daudi cell mitochondria can be achieved, as is evident from the results of the EPR spectra analysis.

Considering the alkyl chain between TPP^+^ and nitroxide moiety, it has been shown that alkyl derivatives of TPP collapse the mitochondrial membrane potential and more effectively inhibit the respiratory chain than the equal concentration of carbonyl cyanide-4-(trifluoromethoxy)phenylhydrazone (FCCP). Moreover, TPP^+^ alkyl derivatives bind nonspecifically to the inner mitochondrial membrane, which results in a loss of membrane integrity and causes a breakdown of its insulating properties and impairment of the phospholipid milieu faced by respiratory complexes [***24***]. Thus, in addition to its selective localization toward cancer cell mitochondria, Mito-CP exhibits inhibitory effects on mitochondrial bioenergetics, as evidenced by studies on membrane potential and cellular ATP levels. Similar effects of Mito-CP on inhibiting the membrane potential of medullary carcinoma cell line has been shown [***25***].

The antioxidant property of Mito-CP involves both superoxide dismutase (SOD) and catalase mimetic activity. It essentially takes place in two steps: 1) conversion of superoxide (O_2_-) into H_2_O_2_ by Mito-CP [***16***], and 2) activation of catalase mimetic activity of heme proteins such as metmyoglobin (an Fe(III) form of myoglobin) by nitroxides resulting in H_2_O_2_ dismutation accompanied by O_2_ evolution [***26***]. In normoxic conditions, the levels of metmyoglobin are limited; thus, the slow-but-steady accumulation of H_2_O_2_ could occur by the SOD activity of Mito-CP. This could account for the absence of changes in the H_2_O_2_ levels in Daudi cells treated with Mito-CP under normoxia in our study. However, the significant reduction of H_2_O_2_ levels after Mito-CP treatment under hypoxia can be correlated with the increased drug localization and levels of myoglobin under hypoxia [***27***]. In PBMCs, the effect of Mito-CP on H_2_O_2_ levels was more pronounced due to its high dependency on mitochondrial-coupled respiration for ROS generation rather than NOX2-based ROS, which is elevated in Daudi cells. In this regard, inhibition of the electron flow itself has been shown to decrease ROS generation under conditions where NOX2-based ROS is not dominant. Thus, the effect of Mito-CP on cellular H_2_O_2_ levels in PBMCs could be attributed to its combined effect on mitochondrial electron transport and its direct antioxidant scavenging properties. Moreover, the antioxidant scavenging by Mito-CP involves the generation of oxygen, which in turn could support the mitochondrial-dependent ATP synthesis as observed from our study on cellular ATP levels. This also corroborates earlier findings on the effect of Mito-CP in enhancing and irreversibly reducing oxygen consumption in normal and cancerous breast cancer cells, respectively [***28***].

Cellular ATP levels have been shown to play a decisive role in cell survival signaling mediated by Akt and AMPK phosphorylation. A Decrease in ATP levels have been shown to increase the activation phosphorylation of AMPK and subsequently decrease the activation phosphorylation of Akt [***29***]. Dephophorylation of Akt is done by two types of phosphatases, viz., PP2A and PHLPP1/2 (PH domain leucine rich repeat protein phosphatase). In conditions of availability, ATP competitively binds to phosphorylated Akt and forms a phosphatase-shielding cage. This prevents phosphatase from executing its dephosphorylation reaction on Akt. Thus, ATP availability sustains Akt-mediated survival and apoptosis inhibition, even in the presence of active phosphatases [***30–32***]. In our study, we found that Mito-CP treatment decreased the phosphorylation of Akt and increased the phosphorylation of AMPK, which correlated with the decrease in ATP levels in Daudi cells under both hypoxia and normoxia. However, Mito-CP treatment in PBMCs did not affect the phosphorylation of Akt and AMPK, which correlated with its negligible effects on ATP levels.

In addition to ATP, an elevated ROS response is known to influence the Akt-mediated growth/survival pathway by its redox reaction with PP2A [***33***]. A key feature of this regulation is that PP2A’s activity on Akt also requires reduced ATP levels to overcome the phosphatase-shielding cage formed by ATP, as discussed previously. Thus, decreasing the ROS levels (such as H_2_O_2_) alone is not a viable option for inhibiting cancer cell survival and inducing apoptosis unless it is coupled with inhibition of ATP production. Interestingly, Mito-CP fits this dual mode of action in cancer cells by decreasing H_2_O_2_ levels via its antioxidant nitroxide moiety and reducing mitochondrial ATP production via its alkyl TPP^+^ moiety. Moreover, Mito-CP’s inhibitory effect on hypoxic adaptations in cancer cells (such as aerobic glycolysis mediated by HIF-1α [***34, 35***] can be correlated with its effects on Akt and H_2_O_2_, which are required for synthesis and stabilization of HIF-1α, respectively [***36, 37***]. This suggests a novel mode of action of Mito-CP in influencing the cancer survival signaling under both normoxic and hypoxic conditions in Daudi cells mediated by EBV.

Direct support for this dual mechanism of Mito-CP comes from an earlier study with Dec-TPP, a control compound for Mito-CP, which lacks the carboxy proxyl moiety and, hence, the antioxidant property. It has been shown that Dec-TPP exhibits significant ATP reduction without inducing cytotoxicity in breast cancer cells [***28***]. However, a study comparing the effects Mito-CP and Mito-CP-acetamide, the nitroxide moiety of which had been modified into an acetamide, did not show any difference in cytotoxicity in pancreatic cancer cells [***38***]. Though not supporting a direct antioxidant mechanism, this study suggests an indirect antioxidant mechanism via NOX inhibition by a mitochondrially targeted antioxidant [***39***], which was significant under conditions of cisplatin-induced oxidative stress but not under normal conditions [***40***]. Considering that acetamide derivatives have been shown to possess antioxidant properties [***41***] and NOX activity itself is directly activated by Akt-mediated phosphorylation [***42***]. Our studies with Dec-TPP also supports the essential role of antioxidant moiety for cytotoxic action of Mito-CP in Daudi cells. The dual mode of action of Mito-CP proposed in this work directly adds support to the above findings on Mito-CP’s mechanism of cancer cytotoxicity.

Further, we confirmed that the Akt-mediated survival signaling cascade toward apoptosis regulation was also influenced by Mito-CP in Daudi cells under both hypoxia and normoxia. Specifically, Mito-CP caused a downregulation of the antiapoptotic factor XIAP; caused an upregulation of BAX expression and protein levels of cytochrome C; and cleaved PARP, which eventually contributed to apoptosis in Daudi cells. This mechanism was validated by treating Daudi cells and PBMCs with the Akt pathway inhibitor wortmannin, which showed similar effects as that of Mito-CP with respect to Akt—S473 phosphorylation and cleaved PARP levels.

**Fig 7:**
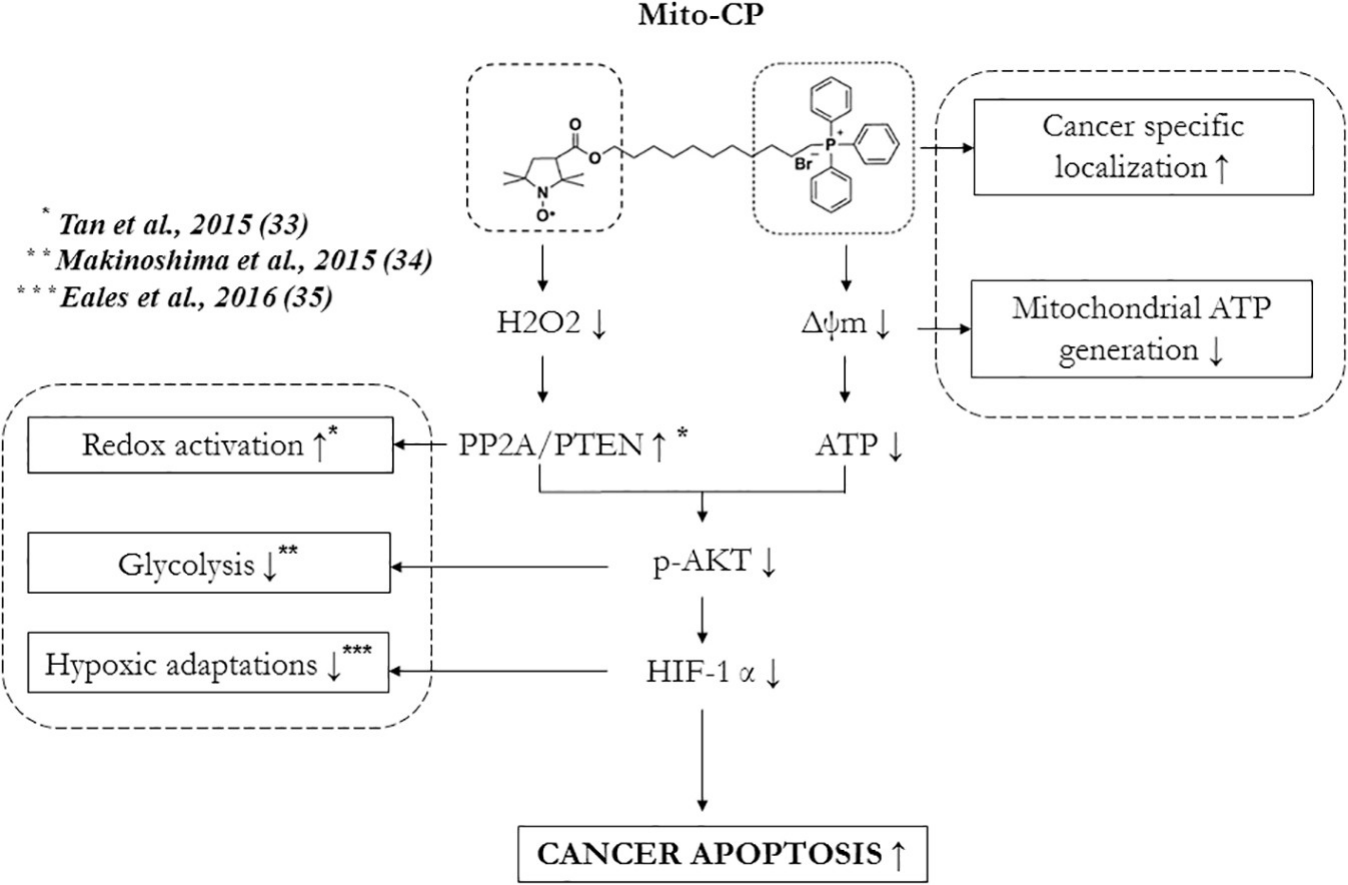
Schematic representation of mechanism of anticancer activity of Mito-CP in Daudi cells.

In conclusion, this study has shown for the first time that Mito-CP selectively induces apoptosis in Daudi cells by mitochondrial potential dependent localization and downregulation of survival strategies mediated by Akt. The effects of Mito-CP on Akt in Daudi cells required both its antioxidant properties and mitochondrial bioenergetics ***"Fig 7”***. This prompts the possibility for its evaluation and therapeutic application in cancers that arise from viral or mutagenic transformations similar to that occurring in Daudi cells by EBV. Moreover, Mito-CP can be evaluated for its suppression of EBV reactivation, which is associated with pathologies (such as infectious mononucleosis) and autoimmune diseases (such as rheumatoid arthritis and multiple sclerosis) and translated clinically in combination with other antiviral drugs.
